# Lateral-stabilized total knee arthroplasty enables design-specific kinematic behavior consistent with key physiological characteristics during flexion

**DOI:** 10.3389/fbioe.2026.1804325

**Published:** 2026-07-24

**Authors:** Philippe Moewis, Rainald Ehrig, Leonie Krahl, Adam Trepczynski, Jonas Tykfer, Hagen Hommel, Georg N. Duda

**Affiliations:** 1 Berlin Institute of Health at Charité – Universitätsmedizin Berlin, Julius Wolff Institute, Berlin, Germany; 2 Zuse Institut Berlin, Berlin, Germany; 3 implantcast GmbH, Buxtehude, Germany; 4 Krankenhaus Märkisch-Oderland GmBH, Wriezen, Germany

**Keywords:** anterior knee pain, fluoroscopy, knee joint biomechanics, knee joint kinematics, total knee arthroplasty

## Abstract

**Introduction:**

Anterior knee pain (AKP) remains as one of the most cited causes of patient’s dissatisfaction after total knee arthroplasty (TKA) with non-physiological knee joint kinematics patterns during flexion and patellar maltracking being frequently associated with it. Incorporating *in vivo* knee joint kinematics into the design of new TKA systems could significantly reduce these negative outcomes. This study aims to show the outcome of a new TKA system developed considering previously assessed *in vivo* knee joint kinematics. Retrospective study design.

**Materials and methods:**

Eleven patients treated with the 5C® LATic TKA system were included. *In-vivo* fluoroscopic measurements in loaded lunge to collect the 3D TKA motion from extension to maximal flexion was conducted at 12 months after index surgery. All patients answered the KSS, FJS and HFKS questionnaires.

**Results:**

The kinematics of the 5C® LATic system were characterized by a lateral stability as well as reduced anterior movement in the medial compartment, resulting in an external rotation of the femoral component on the tibia plateau. Moreover, the frontal points reveal a consistent posterior movement of the frontal aspect until maximal achieved flexion. All patients showed improvements in all administered questionnaires compared to their preoperative state, with high overall satisfaction reported for the prosthesis.

**Discussion:**

The results show an expected design-based kinematics. Although the posterior translation of the frontal aspect of the femoral component can only be interpreted as an indirect measure of patellar movement, the increased motion observed, together with the external rotation on the tibia plateau, may contribute to improvement in patellar tracking, which is recognized as a direct contributor to AKP.

## 1 Introduction

Total knee arthroplasty (TKA) is an effective therapy for end stage knee osteoarthritis (OA), reliably reducing pain and improving function. However, around 20%–25% report dissatisfaction with the outcome (Agarwala, Mohrir, and Patel, 2013; Duan et al., 2018; Feczko et al., 2017; Gunaratne et al., 2017; Hamilton et al., 2015; Hoveidaei et al., 2024; Konno et al., 2014; Laubach et al., 2020; Selvanathan, Ayeni, and Sorial, 2024), although more recent studies indicate a reduction to approximately 10% (Ayers et al., 2022; DeFrance and Scuderi, 2023; Kahlenberg et al., 2016).

One of the most cited reasons for dissatisfaction, often leading to early revision surgery, is anterior knee pain (AKP), reported in 4%–40% of cases (Agarwala, Mohrir, and Patel, 2013; Antinolfi et al., 2020; Breugem et al., 2008; Duan et al., 2018; Feczko et al., 2017; Feng et al., 2024; Koh et al., 2019; Konno et al., 2014; Laubach et al., 2020; Lee et al., 2013; Park et al., 2016; Petersen et al., 2014; Popovic and Lemaire, 2003; van Houten, Heesterbeek, and Wymenga, 2016; Waters and Bentley, 2003). Although AKP is multifactorial (Feczko et al., 2017), non-physiological knee joint kinematics during flexion and postoperative patellar maltracking are frequently given as underlying reasons (Duan et al., 2018; Lee et al., 2013).

**FIGURE 1:**
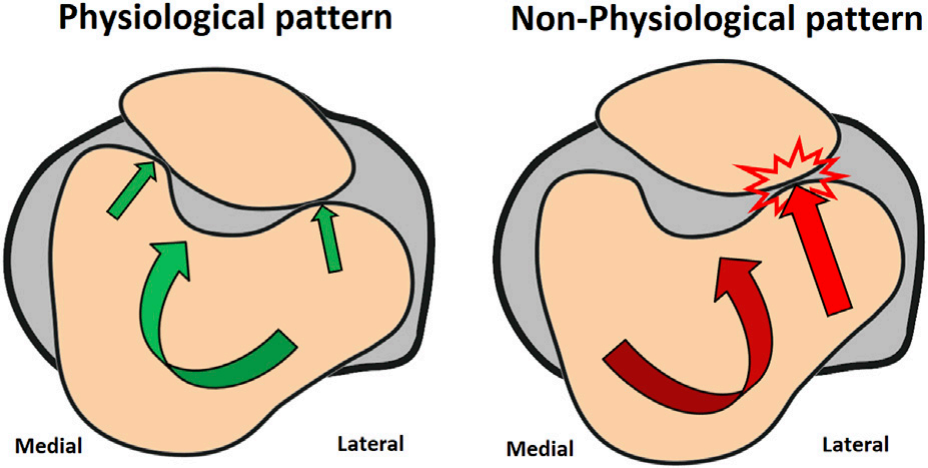
Illustration of physiological (left) and non-physiological axial rotational patterns (right) during knee flexion.

Physiological knee kinematics combine lateral femoral rollback and medial pivot, which results in external rotation of the femur (Pinskerova et al., 2009). This pattern is crucial for proper knee flexion, as it reduces excessive shear forces on the patellofemoral joint—particularly on its lateral side—and contributes to balanced patellar tracking, thereby lowing the risk for AKP. In contrast, non-physiological axial femoral rotation may disrupt the medial pivot pattern, destabilize the lateral compartment, and increase lateral patellofemoral pressure during deep flexion (Figure 1). This, in turn, could elevate the risk of AKP (Konno et al., 2014).

An *in vivo* study conducted at our institution involving 10 healthy knees (mean age 35.8 years) during weight-bearing lunge showed a similar kinematic pattern, albeit with reduced lateral femoral rollback (Moewis et al., 2023).

Despite the introduction of various design variations such as posterior stabilized (PS) and cruciate retaining (CR), inconsistent anterior-posterior (AP) translation and reversed axial rotation during deep flexion persist (Bailey et al., 2015; Jiang et al., 2016; Moskal and Capps, 2014; Zingde and Slamin, 2017). Asymmetrical TKA designs were developed to restore physiological patterns, yet instability in both compartments has been reported (Moewis et al., 2021). On the other hand, an *in vivo* fluoroscopic study of 10 TKA patients at our institution showed a stable lateral compartment but reduced axial translation on the tibial plateau (Moewis et al., 2019).

Given these contradictory findings, a novel TKA design concept was developed incorporating *in vivo* knee kinematics into implant development. We hypothesized that targeted design modifications could enable specific motion patterns, even under loaded deep flexion situations. In this study, we focus on the optimization of a lateral-ventral stabilized TKA design. The primary objective of the present study is not to demonstrate the superiority over conventional TKA systems, but rather to determine if the intended design modification would result in the expected kinematic behavior. Understanding the effects of this specific TKA design adaptation could pave the way to more personalized TKA selection to appropriately match the needs of patients suffering from knee OA.

## 2 Materials and methods

### 2.1 TKA implant description

The TKA system presented in this study, the 5C® LATic, was developed based on results from previous *in vivo* fluoroscopic measurements conducted at our institution in a cohort of 10 patients (mean age: 66.1 years; six females, four males) with asymmetrical lateral-stabilized knee implants (3DKnee CR; DJO Surgical Inc.) during weight-bearing lunge activity. As described in the Introduction, a stable lateral compartment but reduced axial translations on the tibial plateau were observed from this analysis (Moewis et al., 2019). Details regarding the *in vivo* fluoroscopic data collection and post-processing will be further explained in the Data Acquisition and Data Post-Processing sections.

**FIGURE 2:**
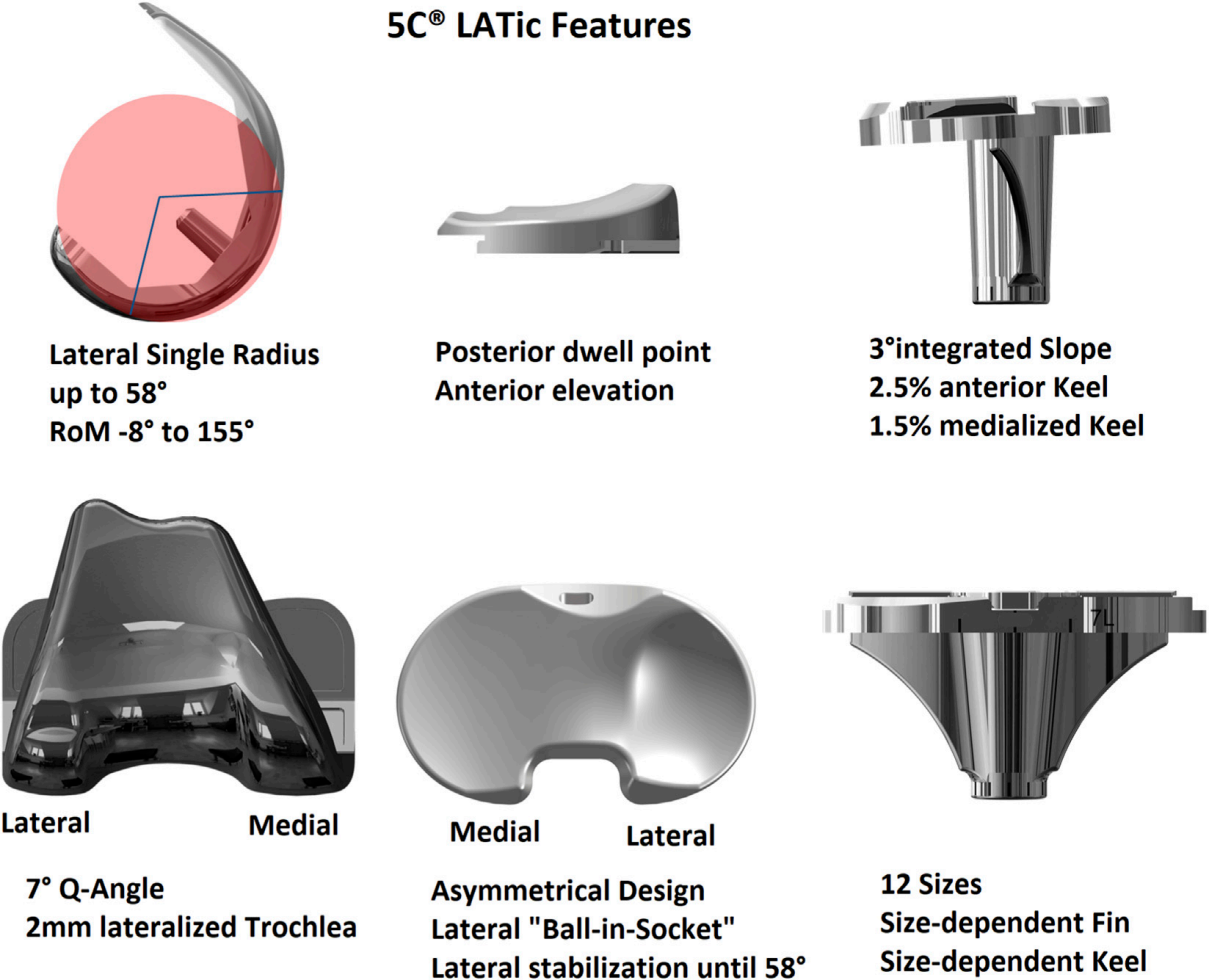
Overview of the main geometrical features of the 5C® LATic TKA system.

The 5C® LATic is a cruciate-retaining (CR) system with an asymmetric design aimed at promoting defined and reproducible motion patterns during flexion. To enhance lateral stability and facilitate an external axial rotation pattern of the femoral component, its bearing geometry features a flat medial compartment and a congruent, ball-in-socket lateral compartment, which remains engaged until 58° of knee flexion. An anterior elevation is incorporated to reduce paradoxical anterior shift in the lateral compartment. Additionally, the femoral component employs a single sagittal radius up to 58° of knee flexion, transitioning to a multi-radius design beyond this point to facilitate femoral rollback during deeper flexion. To optimize patellar tracking, the frontal aspect of the femoral component incorporates a 7° Q-angle and a 2 mm lateralized trochlea. The sulcus angle extends to 165.5° at 0° of knee flexion and narrows to 157.1° at 45° of knee flexion (Figure 2).

### 2.2 End stage OA patients treated with the 5C® LATic system

**FIGURE 3:**
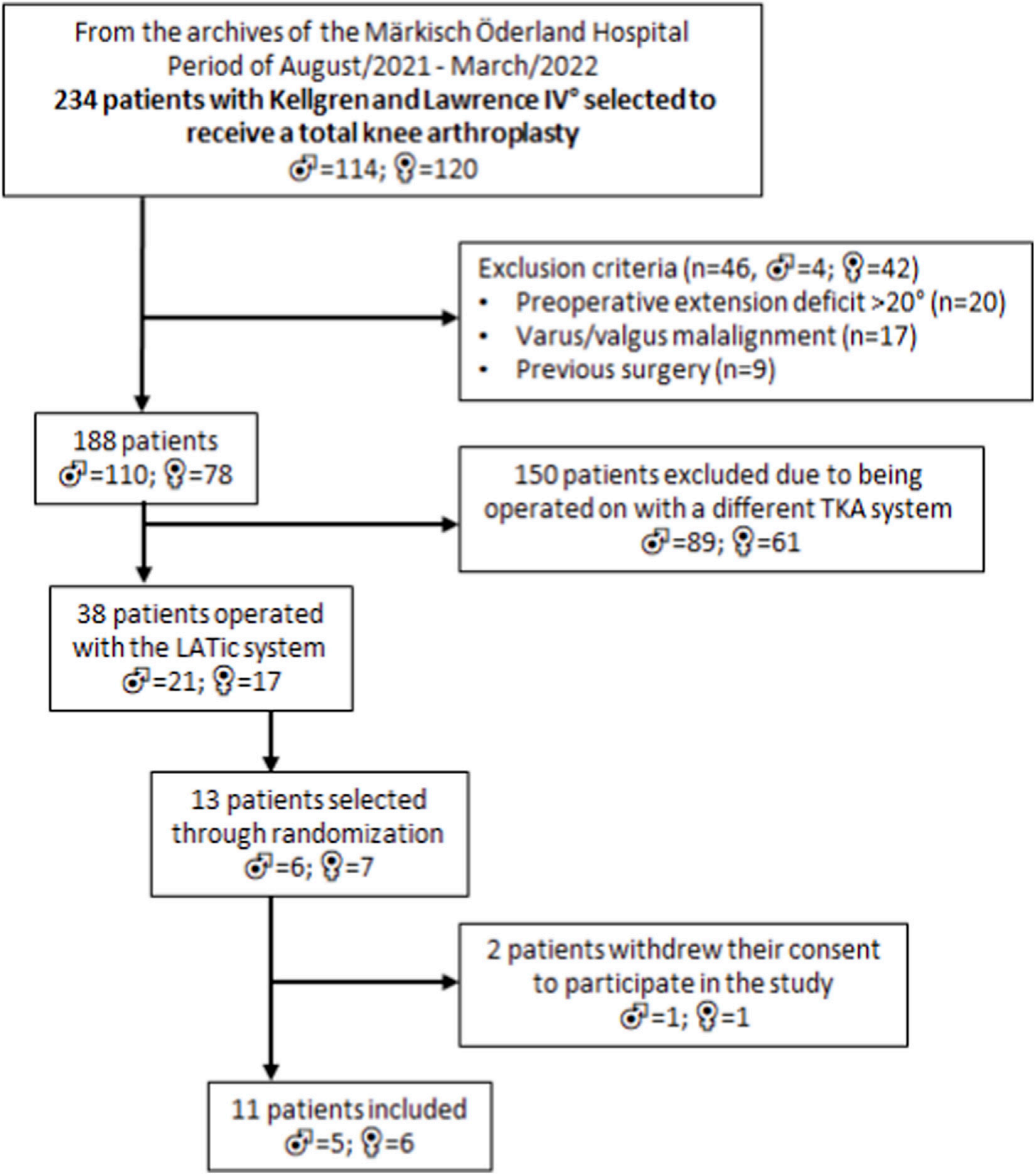
CONSORT Diagram for the 5C® LATic TKA cohort.

Eleven patients (mean age: 70.5 years, six females, five males) were randomly selected to receive the 5C® LATic TKA system (implantcast GmbH). Baseline demographic characteristics (age, sex, body mass index (BMI) and operated side) are summarized in Table 1. The primary inclusion criteria being a diagnosis of osteoarthritis, a coronal deformity of <10°, and a clear indication for TKA. Exclusion criteria included a preoperative extension deficit of >20°, valgus/varus malalignment, and prior surgery on the affected joint (see CONSORT [Consolidated Standards of Reporting Trials] diagram in Figure 3). Measurements were conducted at a minimum of 12 months post-surgery. The study was approved by the local ethics committee (Landesärztekammer Brandenburg, Germany, approval No: S10 [a]/2018) and registered in the German Clinical Trials Register (DRKS00010775). All participants provided written informed consent. The aforementioned cohorts analyzed at our institution, comprising ten healthy knees and ten knees treated with the 3DKnee CR design, were used for comparison.

**TABLE 1:** Demographic data of the 5C^®^ LATic cohort.

Patient	Age (yr)	Sex	BMI (kg/m2)	Operated side
1	66	W	32.6	Right
2	74	M	27.3	Right
3	83	M	30.1	Left
4	71	W	31.5	Left
5	71	W	27.2	Left
6	63	W	32.8	Left
7	63	M	28.6	Left
8	67	M	29.8	Left
9	76	M	30.5	Right
10	72	W	31.9	Left
11	70	W	34.5	Left
Mean	70.5	-	30.6	​

### 2.3 Surgical technique

All operations were performed by a single surgeon (HH). General endotracheal anesthesia and periarticular injection were administered, and a tourniquet was used in all cases. A medial parapatellar approach was employed for all exposures (Hommel, Abdel, and Perka, 2017). Flexion and extension gap balancing was achieved using the “extension gap-first technique” with a specialized balancing device (Hube et al., 2011). The posterior cruciate ligament (PCL) was preserved. The femur was distracted from the proximal tibia using the balancer device (Hommel, Abdel, and Perka, 2017).

With the adjusted mechanical alignment technique, the goal of implant positioning was to achieve equal tension in the medial and lateral collateral ligaments, rather than strictly aiming for a neutral axis (Hommel, Abdel, and Perka, 2017; Hommel and Perka, 2015; Hommel, Perka, and Pfitzner, 2016). Distal femoral alignment was modified using a medial varus correction cut of up to 2.5° using an adjustable cutting block. After reassessment of the extension gap, femoral preparation proceeded once symmetrical gap balance was achieved.

The established values for the extension gap were then applied to the flexion gap. Femoral rotation was guided by the tension of the soft tissues to create a rectangular flexion gap. Final bone cuts were made, and the implant was placed as usual (Hommel, Abdel, and Perka, 2017).

All patients followed the same postoperative rehabilitation pathway, which consists of a 5-day inpatient hospital stay according to the institutional arthroplasty protocol (Krankenhaus Märkisch-Oderland GmBH, Wriezen, Germany), followed by a standardized 3-week inpatient rehabilitation program at the affiliated rehabilitation center in Bad Freienwalde, Germany. These procedures were implemented to reduce variability related to surgical technique and postoperative care.

### 2.4 Data Acquisiton at a minimum of 12 months post-surgery in patients with the 5C® LATic system

Single-plane fluoroscopic analysis was conducted at the Julius Wolff Institute, Charité-Universitätsmedizin Berlin, using a Philips BV Pulsera fluoroscope (Philips Medical Systems) to capture knee kinematics with a pulsed imaging sequence at an acquisition frequency of 30 Hz (Moewis et al., 2012). To correct image distortion, additional images were collected using a specially designed Perspex calibration box with 553 fiducial markers and 45 control markers (Garling et al., 2005). Patients were asked to perform a single-limb weight-bearing lunge to facilitate analysis of tibiofemoral kinematics in challenging knee flexion scenarios, a single-limb non-weight-bearing knee flexion-extension was additionally conducted to understand the effect of the geometry with reduced influence of the loading in the knee joint.

**FIGURE 4:**
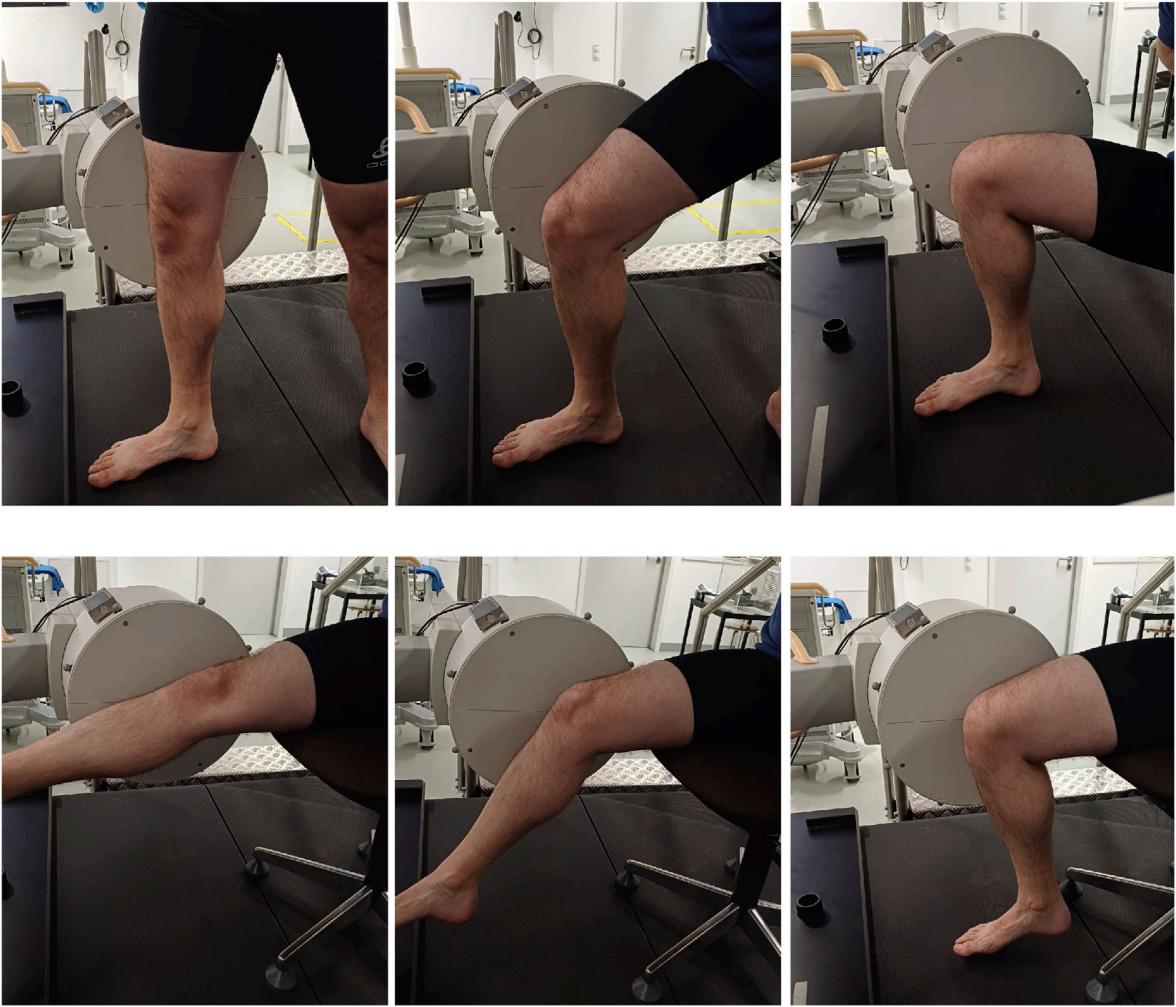
Visualization of the weight-bearing lunge activity (top) and the non-weight-bearing flexion-extension, from extension to maximum flexion.

Care was taken to clearly explain the activities to subjects and ensure proper execution, thereby minimizing errors and unnecessary radiation exposure during the fluoroscopic analysis. For the lunge, both feet were positioned level on a platform, with the contralateral leg placed further back to avoid overlap (Figure 4, Top). The flexion–extension task was performed in a seated position (Figure 4, Bottom). Both activities began at full knee extension, moved through maximal knee flexion, and concluded by returning to full extension. Each patient completed three repetitions of each activity (Moewis et al., 2021; Moewis et al. 2019; Moewis et al. 2023; Pfitzner et al., 2017). Following fluoroscopic imaging acquisition, the complete image sequences were exported in DICOM (Digital Imaging and Communication in Medicine) format and subsequently converted into individual high-quality BMP (Bitmap Image File) images for further analysis.

### 2.5 Clinical data and questionnaires of patients with the 5C® LATic system

The following clinical data were collected: preoperative and postoperative knee range of motion; clinical questionnaire scores, including the Knee Society Score (KSS) Knee Score (KS) and Function Score (FS) (Ewald, 1989), the Forgotten Joint Score (FJS) (Behrend et al., 2012), and the High Flexion Knee Score (HFKS) (Na et al., 2012). Postoperative subjective patient satisfaction was also assessed. Data were collected by an independent physiotherapist who was not involved in the surgical procedures. All the clinical information is reported in Table 8.

### 2.6 Data Post-processing and analyses

**FIGURE 5:**
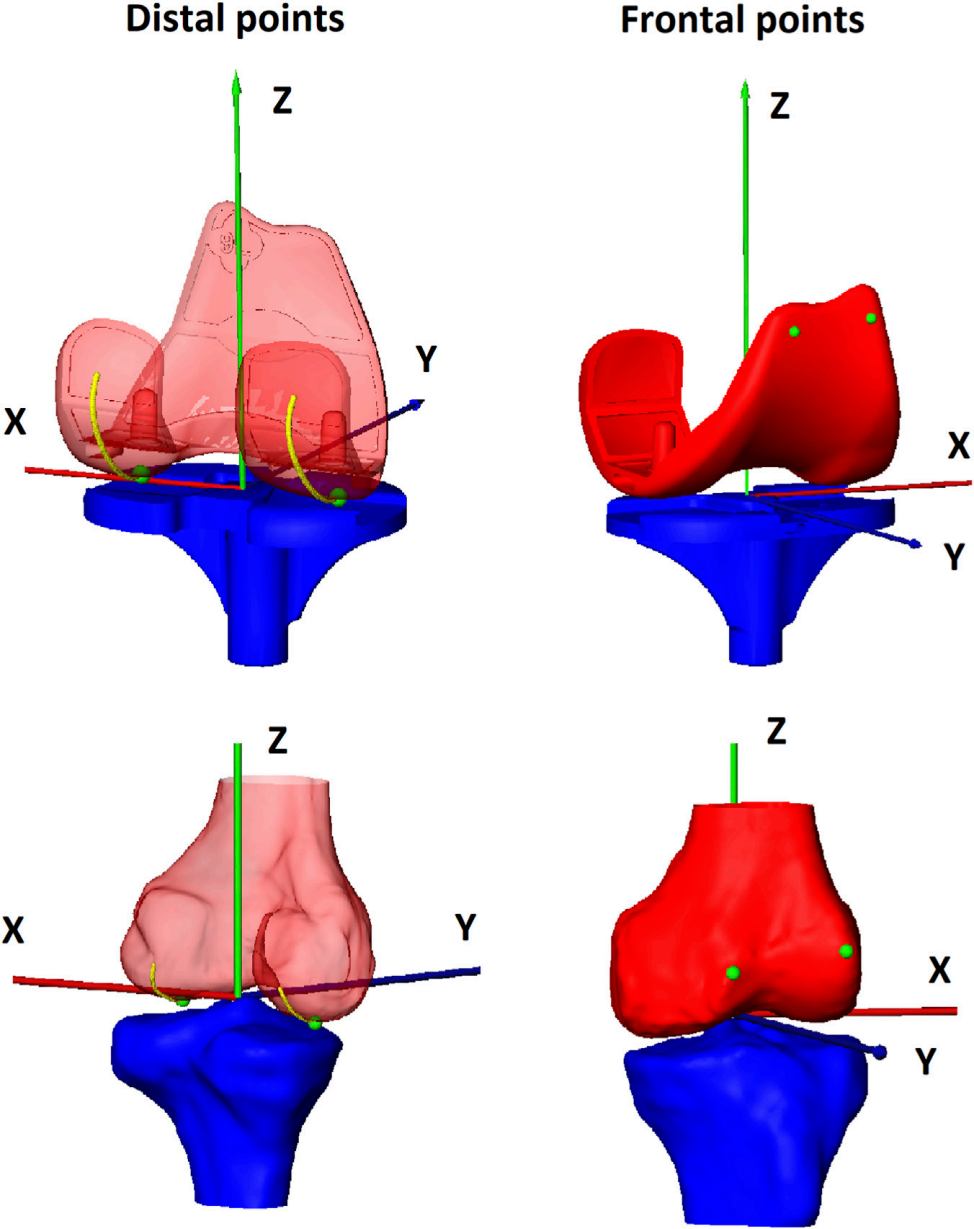
Identification of distal and frontal points on TKA components (top) and native knees (bottom) for the quantification of tibiofemoral kinematics.

Fluoroscopic BMP images were selected at 5° flexion intervals between maximal extension and maximal flexion (Moewis et al., 2021; Moewis et al. 2019; Moewis et al. 2023). High-quality three-dimensional (3D) computer-aided design (CAD) models of the femoral and tibial metallic components of the 5C® LATic system were provided by the manufacturer (implantcast GmbH). The origin of the coordinate system on the tibial component was defined as the intersection of the tibia plateau and the longitudinal axis of the tibial stem. The Z-axis was aligned to the stem axis and pointed proximally, the X-axis represented the medio-lateral direction and pointed to the left and the Y-axis represented the anterior-posterior direction and pointed anteriorly. The femoral and tibial CAD models were then registered to the fluoroscopic images using the Model-Based RSA software (RSA Core, MEDIS Medical Imaging Systems, Leiden, Netherlands). As single-plane fluoroscopy is an indirect measurement technique, the accuracy of the calculated joint kinematics depends on a proper contour detection. The registration procedure consisted of an initial automatic contour detection followed by independent manual corrections to refine relevant contours and discard erroneously detected contours segments. The systematic error associated with the automatic contour detection procedure has been evaluated previously in a phantom experiment and was found to be below 0.1 mm and 0.1° (Prins et al., 2011). Furthermore, the accuracy of the complete registration procedure under dynamic conditions has been also evaluated, showing errors of 0.2–0.6 mm for in-plane translations and 0.4°–0.8° for rotations (Moewis et al., 2012). Following the 3D registration process, the positions and orientations of the femoral and tibial components were used to identify the most distal points of the lateral and medial femoral condyles and their projections on the tibial component plateau (Figure 5, Top). The positional changes of both distal points throughout the knee flexion cycle were used to determine the anterior-posterior translation of the medial and lateral femoral condyles, as well as the axial rotation relative to the tibial component.

In addition to the conventional analysis of tibial plateau movement conducted in previous studies (Moewis et al., 2021; Moewis et al. 2019; Moewis et al. 2023; Moewis et al. 2012), a frontal plane perpendicular to the tibial component plateau was defined at its most anterior position to identify the anterior tangential frontal points on the femoral components. These frontal reference points were specifically selected because they correspond to the region of the femoral component interacting with the patellofemoral mechanism and therefore may serve as an indirect surrogate of patellar motion throughout the entire flexion cycle. This analysis was also applied to the available ten healthy knees, where the frontal points were determined on the femoral bone, and to ten knees implanted with the 3DKnee CR design (Figure 5, Bottom). A control group with a more conventional TKA system was not included because the primary endpoint was the verification of design-specific kinematic rather than a comparative clinical performance. To compare outcome values across different trials and generate mean values at identical knee flexion angles, data were resampled using linear interpolation at uniform 1° flexion increments.

### 2.7 Statistical analysis

Data are reported as mean ± standard deviation. Normality was assessed using the Kolmogorov-Smirnov test. Fluoroscopic data were analyzed using the Mann-Whitney U Test. Statistical significance was defined as p < 0.05. All analyses were performed using SPSS software (version 30; IBM, Armonk, NY, United States of America). A postr hoc power analysis was conducted to estimate the sample sizes required to achieve a statistical power of 1−β = 0.80 with an alpha of 0.05. Based on the magnitude of anterior-posterior translation as the primary outcome parameter derived from previous investigations, sample sizes of ×10 10 for the comparison between 5C® LATic TKA knees and healthy knees; and of 6 x 6 for the comparison between 5C® LATic TKA knees and 3DKnee TKA knees were determined.

## 3 Results

### 3.1 Fluoroscopic analysis

**FIGURE 6:**
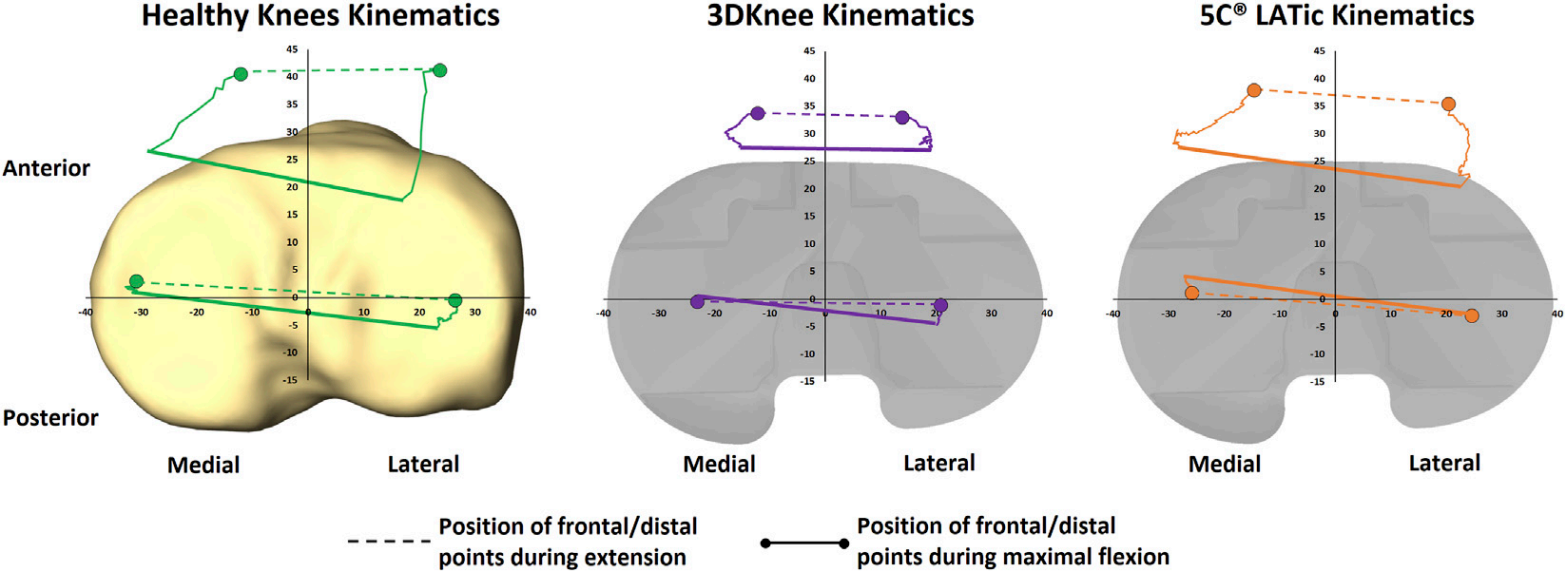
Frontal and distal kinematics (mean values) of the medial and lateral compartments during a weight-bearing lunge activity in knees treated with the 5C® LATic TKA system (right), compared to those in healthy cohorts (left) and knees implanted with the 3DKnee TKA system (middle).

**FIGURE 7:**
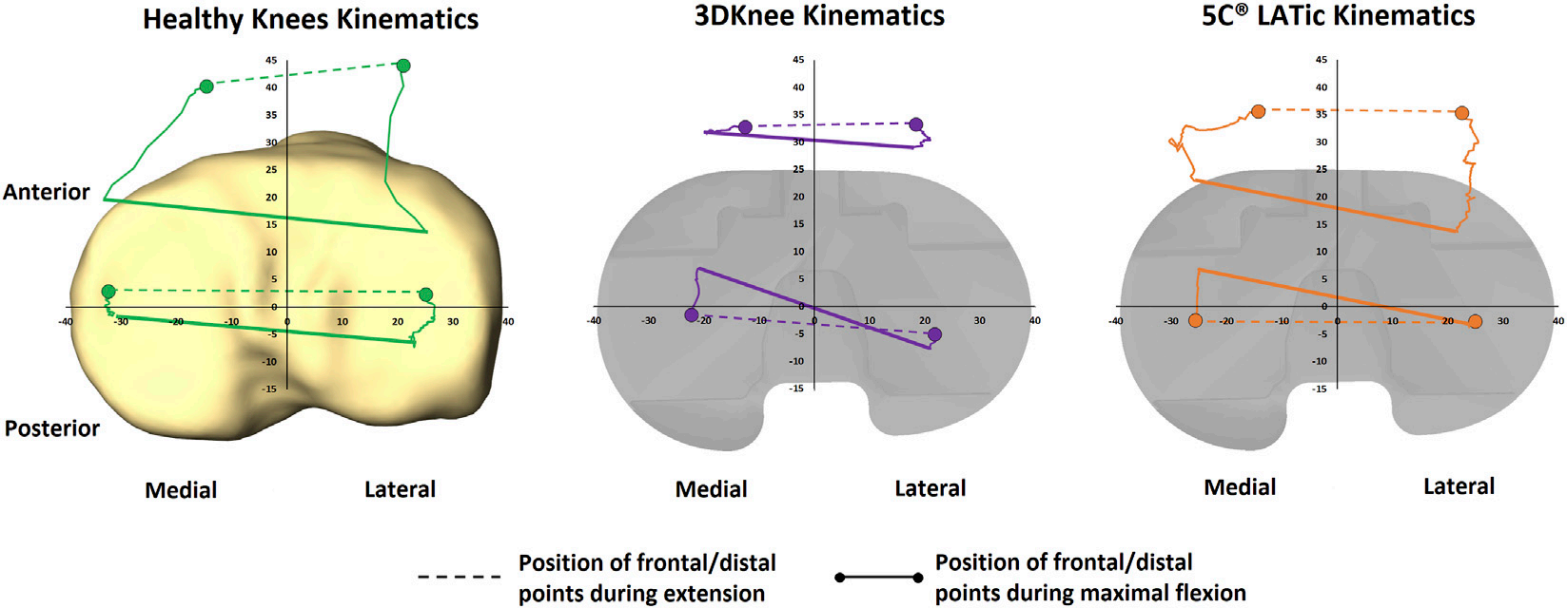
Frontal and distal kinematics (mean values) of the medial and lateral compartments during a non-weight-bearing flexion-extension activity in knees treated with the 5C® LATic TKA system (right), compared to those in healthy cohorts (left) and knees implanted with the 3DKnee TKA system (middle).

During the weight-bearing lunge, the anterior-posterior translation, derived from the changes in the absolute position of the distal points on the tibia plateau as well as the frontal points on the femoral bone/components, the healthy cohort exhibited stable motion in the medial compartment movement and certain degree of lateral rollback. In contrast, both medial and lateral frontal points showed a continuous posterior translation. The 3DKnee cohort was characterized by a stable medial compartment and reduced lateral rollback on the tibia plateau, accompanied by a clearly reduced posterior movement of the frontal points. The 5C® LATic cohort demonstrated design-based lateral stability and reduced anterior movement in the medial compartment. However, the movement of the anterior frontal points revealed a clear and consistent posterior movement of the frontal aspect of the femoral component from full extension to maximum achieved flexion. This pattern was particularly pronounced on the lateral side. A graphical representation of this cohort-specific kinematic patterns is provided in Figure 6. Notably, the posterior movement was even more pronounced in the lateral compartment during the non-weight-bearing flexion-extension activity (Figure 7).

#### 3.1.1 Anterior-posterior translation on the tibia plateau

Considering the magnitude of the motion on the tibia plateau during the weight-bearing lunge, calculated relative to the first fluoroscopic frame, the maximum medial compartment translation in the 5C® LATic cohort was 2.5 ± 2.1 mm (anterior direction), compared to 2.6 ± 5.0 mm in the healthy knees cohort (*p* = 0.95) and 0.8 ± 1.2 mm in the 3DKnee cohort (*p* = 0.02). On the lateral compartment, the 5C® LATic cohort remained relatively stable with a posterior translation of −0.7 ± 1.7 mm compared with a significantly greater posterior translation of −5.0 ± 4.7 mm in the healthy cohort (*p* = 0.02) and −4.5 ± 3.0 mm in the 3DKnee cohort (*p* = 0.004). The complete motion in both compartments across the full range of flexion is summarized in Table 2. During the non-weight-bearing flexion-extension the maximum medial compartment translation in the 5C® LATic cohort was 7.9 ± 2.9 mm (anterior direction) compared to a significant posterior translation of −2.5 ± 5.5 mm in the heathy knees cohort (*p* = 0.001) and to anterior translation of 6.0 ± 3.3 mm in the 3DKnee cohort (*p* = 0.20). On the lateral compartment, the 5C® LATic cohort remained also stable with a posterior translation of −0.7 ± 2.2 mm compared to significantly higher posterior translation of −10.7 ± 7.2 mm in the healthy knees cohort (*p* = 0.001) and to −2.8 ± 1.6 mm in the 3DKnee cohort (*p* = 0.08). The complete motion in both compartments across the full range of flexion is summarized in Table 3.

**TABLE 2:** Means and standard deviations of the relative anterior-posterior kinematics of the distal points during weight-bearing lunge for the 5C^®^ LATic, Healthy and 3DKnee cohorts.

​		Medial compartment					Lateral compartment				
		5C® LATic (mm)	Healthy (mm)	Effect size (p-value)	3DKnee (mm)	Effect size (p-value)	5C® LATic (mm)	Healthy (mm)	Effect size (p-value)	3DKnee (mm)	Effect size (p-value)
**Knee Flexion Angle (°)**	0	0	0	-	0	-	0	0	-	0	-
	10	0.5±0.4	-1.2±2.1	1.09 (0.12)	0.1±0.4	0.81 (0.08)	**0.1±0.2**	**-3.6±3.8**	1.38 (0.03)	**-0.3±0.3**	1.39 (0.004)
	20	**1.4±0.8**	**-2.3±3.0**	1.62 (0.007)	**0.5±0.7**	1.08 (0.03)	**0.3±0.4**	**-6.3±5.6**	1.67 (0.002)	**-0.4±0.7**	1.23 (0.01)
	30	**2.5±1.5**	**-3.3±3.0**	2.44 (0.001)	**1.1±1.0**	1.07 (0.02)	**0.5±0.7**	**-6.4±5.8**	1.66 (0.001)	**-0.4±0.9**	1.13 (0.04)
	40	**3.5±1.9**	**-4.5±3.7**	2.72 (0.001)	**1.8±1.3**	1.03 (0.02)	**0.6±1.0**	**-6.9±5.5**	1.90 (0.001)	**-0.5±0.9**	1.06 (0.04)
	50	**4.6±2.3**	**-4.6±4.4**	2.60 (0.001)	**2.4±1.5**	1.10 (0.02)	**0.7±1.3**	**-7.2±5.8**	1.86 (0.001)	**-0.7±0.9**	1.16 (0.03)
	60	**6.1±2.2**	**-4.8±4.0**	3.36 (0.001)	**3.0±1.6**	1.62 (0.005)	**0.7±1.4**	**-7.4±6.6**	1.70 (0.001)	**-1.0±0.9**	1.41 (0.01)
	70	**7.2±2.2**	**-4.4±4.1**	3.46 (0.001)	**3.6±1.7**	1.79 (0.002)	**0.9±1.4**	**-7.7±6.0**	1.98 (0.001)	**-1.4±1.0**	1.88 (0.001)
	80	**8.0±2.2**	**-4.0±4.3**	3.54 (0.001)	**4.4±2.1**	1.68 (0.002)	**1.0±1.3**	**-8.6±5.7**	2.30 (0.001)	**-1.7±1.3**	2.05 (0.001)
	90	**8.4±2.4**	**-3.3±5.0**	2.95 (0.001)	5.4±2.7	1.15 (0.06)	**0.5±1.5**	**-9.0±6.7**	1.97 (0.001)	**-1.9±1.7**	1.49 (0.001)
	100	**7.9±2.9**	**-2.5±5.5**	2.35 (0.001)	6.0±3.3	0.60 (0.20)	**-0.7±2.2**	**-10.7±7.2**	1.88 (0.001)	-2.8±1.6	1.07 (0.08)

Bold values indicate significant differences. Effect size and p values for the respective comparison are also presented. Since the subjects achieved different levels of active knee flexion, the analysis was limited to 70° of flexion to ensure inclusion of all participants.

**TABLE 3:** Means and standard deviations of the relative anterior-posterior kinematics of the distal points during non-weight-bearing flexion-extension for the 5C® LATic, Healthy and 3DKnee cohorts.

​		Medial compartment					Lateral compartment				
		5C® LATic (mm)	Healthy (mm)	Effect size (p-value)	3DKnee (mm)	Effect size (p-value)	5C® LATic (mm)	Healthy (mm)	Effect size (p-value)	3DKnee (mm)	Effect size (p-value)
**Knee Flexion Angle (°)**	0	0	0	-	0	-	0	0	-	0	-
	10	-0.7±0.5	-0.0±1.0	0.88 (0.17)	-1.0±0.8	0.42 (0.50)	-1.7±0.9	0.5±4.4	0.66 (0.25)	-1.1±0.5	0.75 (0.06)
	20	**-1.9±0.8**	**-0.3±1.1**	1.62 (0.007)	-1.9±1.6	0.16 (0.51)	-2.9±1.8	-1.3±3.2	0.62 (0.16)	-2.0±0.8	0.60 (0.22)
	30	**-3.4±1.2**	**-1.4±1.8**	1.33 (0.007)	-2.5±2.5	0.47 (0.08)	**-5.2±1.8**	-3.1±3.5	0.71 (0.17)	**-2.8±0.9**	1.62 (0.007)
	40	-4.2±1.9	-3.2±2.9	0.43 (0.39)	-2.9±3.3	0.50 (0.12)	**-6.8±2.5**	-6.3±3.3	0.17 (0.36)	**-3.4±1.2**	1.79 (0.003)
	50	-5.1±2.4	-5.4±3.9	0.10 (0.72)	-3.2±3.9	0.56 (0.08)	**-7.5±2.7**	-9.7±3.5	0.69 (0.29)	**-3.9±1.5**	1.66 (0.004)
	60	-5.5±2.5	-7.5±4.3	0.56 (0.21)	-3.8±4.8	0.44 (0.09)	**-8.6±2.9**	**-13.4±3.4**	1.52 (0.008)	**-4.5±1.8**	1.72 (0.004)
	70	-6.2±2.7	-6.7±3.1	0.20 (0.28)	-5.0±6.0	0.24 (0.34)	**-10.1±2.7**	**-17.0±3.3**	2.29 (0.009)	**-5.5±1.9**	1.98 (0.005)

Bold values indicate significant differences. Effect size and p values for the respective comparison are also presented.

#### 3.1.2 Anterior-posterior translation of the frontal aspect of the femoral bone/component

Regarding the magnitude of the motion of the frontal femoral points, the 5C® LATic cohort demonstrated during weight-bearing lunge a posterior displacement of −6.2 ± 2.7 mm in the medial compartment, compared with −6.7 ± 3.1 mm in the healthy knees cohort (*p* = 0.28) and −5.0 ± 6.0 mm in the 3DKnee cohort (*p* = 0.34). In the lateral compartment the 5C® LATic cohort translated posteriorly up to −10.1 ± 2.7mm, compared with a significantly greater posterior translation of −17.0 ± 3.3 mm in the healthy cohort (*p* = 0.009) and significantly smaller translation of −5.5 ± 1.9 mm in the 3DKnee cohort (*p* = 0.005). The complete motion in both compartments across the full range of flexion is summarized in Table 4. During the non-weight-bearing flexion-extension the 5C® LATic cohort demonstrated a posterior displacement of −4.8 ± 3.1 mm in the medial compartment compared to significantly higher −19.7 ± 5.0 mm in the healthy knees cohort (*p* = 0.001) and to −5.3 ± 6.2 mm in the 3DKnee cohort (*p* = 0.95). In the lateral compartment the posterior displacement of the 5C® LATic cohort was −13.6 ± 3.3 mm compared to significantly higher −29.6 ± 3.6 mm in the healthy cohort (*p* = 0.001) and significantly lower −8.6 ± 4.9 mm in the 3DKnee cohort (*p* = 0.02). The complete motion in both compartments across the full range of flexion is summarized in Table 5.

**TABLE 4:** Means and standard deviations of the relative anterior-posterior kinematics of the frontal points during weight-bearing lunge for the 5C® LATic, Healthy and 3DKnee cohorts.

​		Medial compartment					Lateral compartment				
		5C® LATic (mm)	Healthy (mm)	Effect size (p-value)	3DKnee (mm)	Effect size (p-value)	5C® LATic (mm)	Healthy (mm)	Effect size (p-value)	3DKnee (mm)	Effect size (p-value)
**Knee flexion angle (°)**	0	0	0	-	0	-	0	0	-	0	-
	10	−0.7 ± 0.5	−0.0 ± 1.0	0.88 (0.17)	−1.0 ± 0.8	0.42 (0.50)	−1.7 ± 0.9	0.5 ± 4.4	0.66 (0.25)	−1.1 ± 0.5	0.75 (0.06)
	20	**−1.9 ± 0.8**	**−0.3 ± 1.1**	1.62 (0.007)	−1.9 ± 1.6	0.16 (0.51)	−2.9 ± 1.8	−1.3 ± 3.2	0.62 (0.16)	−2.0 ± 0.8	0.60 (0.22)
	30	**−3.4 ± 1.2**	**−1.4 ± 1.8**	1.33 (0.007)	−2.5 ± 2.5	0.47 (0.08)	**−5.2 ± 1.8**	−3.1 ± 3.5	0.71 (0.17)	**−2.8 ± 0.9**	1.62 (0.007)
	40	−4.2 ± 1.9	−3.2 ± 2.9	0.43 (0.39)	−2.9 ± 3.3	0.50 (0.12)	**−6.8 ± 2.5**	−6.3 ± 3.3	0.17 (0.36)	**−3.4 ± 1.2**	1.79 (0.003)
	50	−5.1 ± 2.4	−5.4 ± 3.9	0.10 (0.72)	−3.2 ± 3.9	0.56 (0.08)	**−7.5 ± 2.7**	−9.7 ± 3.5	0.69 (0.29)	**−3.9 ± 1.5**	1.66 (0.004)
	60	−5.5 ± 2.5	−7.5 ± 4.3	0.56 (0.21)	−3.8 ± 4.8	0.44 (0.09)	**−8.6 ± 2.9**	**−13.4 ± 3.4**	1.52 (0.008)	**−4.5 ± 1.8**	1.72 (0.004)
	70	−6.2 ± 2.7	−6.7 ± 3.1	0.20 (0.28)	−5.0 ± 6.0	0.24 (0.34)	**−10.1 ± 2.7**	**−17.0 ± 3.3**	2.29 (0.009)	**−5.5 ± 1.9**	1.98 (0.005)

Bold values indicate significant differences. Effect size and p values for the respective comparison are also presented. Since the subjects achieved different levels of active knee flexion, the analysis was limited to 70° of flexion to ensure inclusion of all participants.

**TABLE 5:** Means and standard deviations of the relative anterior-posterior kinematics of the frontal points during non-weight-bearing flexion-extension for the 5C® LATic, Healthy and 3DKnee cohorts.

​		Medial compartment					Lateral compartment				
		5C® LATic (mm)	Healthy (mm)	Effect size (p-value)	3DKnee (mm)	Effect size (p-value)	5C® LATic (mm)	Healthy (mm)	Effect size (p-value)	3DKnee (mm)	Effect size (p-value)
**Knee flexion angle (°)**	0	0	0	-	0	-	0	0	-	0	-
	10	−0.9 ± 0.6	−0.9 ± 1.3	0.02 (0.92)	−0.8 ± 0.6	0.15 (0.45)	**−1.6 ± 0.8**	−1.6 ± 1.0	0.04 (0.77)	**−0.7 ± 0.3**	1.39 (0.02)
	20	−1.7 ± 0.9	−1.1 ± 1.3	0.51 (0.36)	−1.4 ± 1.1	0.27 (0.51)	**−3.2 ± 1.0**	−3.7 ± 1.7	0.33 (0.62)	**−1.4 ± 0.5**	2.23 (0.001)
	30	−2.4 ± 1.4	−1.8 ± 2.1	0.33 (0.48)	−1.8 ± 1.6	0.43 (0.21)	**−5.4 ± 1.2**	−5.6 ± 1.9	0.16 (0.67)	**−1.8 ± 0.7**	3.47 (0.001)
	40	−2.8 ± 1.8	−4.8 ± 2.3	0.98 (0.07)	−1.8 ± 2.1	0.46 (0.27)	**−7.2 ± 1.5**	**−9.3 ± 1.9**	1.12 (0.03)	**−2.0 ± 1.1**	3.80 (0.001)
	50	**−2.8 ± 2.0**	**−7.9 ± 3.3**	1.83 (0.002)	−1.7 ± 2.7	0.46 (0.27)	**−7.7 ± 1.8**	**−12.9 ± 2.8**	2.22 (0.001)	**−2.1 ± 1.5**	3.24 (0.001)
	60	**−2.5 ± 2.2**	**−11.2 ± 3.6**	2.92 (0.001)	−1.4 ± 3.4	0.36 (0.25)	**−8.2 ± 1.9**	**−16.7 ± 3.2**	3.20 (0.001)	**−2.3 ± 2.0**	2.98 (0.001)
	70	**−2.3 ± 2.1**	**−14.9 ± 3.5**	4.35 (0.001)	−1.4 ± 4.0	0.27 (0.36)	**−8.8 ± 1.9**	**−21.0 ± 3.3**	4.45 (0.001)	**−2.7 ± 2.5**	2.66 (0.001)
	80	**−2.5 ± 2.1**	**−17.9 ± 4.1**	4.60 (0.001)	−2.0 ± 4.6	0.13 (0.51)	**−9.9 ± 2.1**	**−24.9 ± 3.9**	4.72 (0.001)	**−3.9 ± 3.0**	2.25 (0.001)
	90	**−3.2 ± 2.2**	**−19.1 ± 4.9**	4.09 (0.001)	−3.4 ± 5.3	0.04 (0.93)	**−11.5 ± 2.5**	**−27.3 ± 3.5**	5.22 (0.001)	**−5.9 ± 3.6**	1.81 (0.002)
	100	**−4.8 ± 3.1**	**−19.7 ± 5.0**	3.56 (0.001)	−5.3 ± 6.2	0.10 (0.95)	**−13.6 ± 3.3**	**−29.6 ± 3.6**	4.60 (0.001)	**−8.6 ± 4.9**	1.20 (0.02)

Bold values indicate significant differences. Effect size and p values for the respective comparison are also presented.

#### 3.1.3 Axial rotation

Considering the magnitude of axial external rotation during the weight-bearing lunge derived from the distal points, the femoral component in the 5C® LATic cohort externally rotated up to 3.7° ± 3.4°, compared to 7.6° ± 8.2° in the healthy knee cohort (*p* = 0.12) and 7.0° ± 3.4° in the 3DKnee cohort (*p* = 0.19). When the axial external rotation was derived from the frontal points, the femoral component in the 5C® LATic cohort externally rotated up to 4.9° ± 4.9°, compared with a significantly greater axial external rotation of 13.4° ± 0.9° in the healthy knees cohort (*p* = 0.03). In contrast, the 3DKnee cohort exhibited a reduced axial internal rotation of −0.6° ± 14.3° (*p* = 0.63). The complete motion in both compartments across the full range of flexion is summarized in Table 6. During the non-weight-bearing flexion-extension the axial external rotation of the femoral component derived from the distal points in the 5C® LATic cohort was of 9.7° ± 3.5° compared to 8.2° ± 7.1° in the healthy knees cohort (*p* = 0.33) and 11.3° ± 4.0° in the 3DKnee cohort (*p* = 0.56). Derived from the frontal points, the axial external rotation was 10.6° ± 4.9° in the 5C® LATic cohort compared to 9.7° ± 3.5° in the healthy cohort (*p* = 0.77) and 5.1° ± 18.2° in the 3DKnee cohort (*p* = 0.79). The complete motion in both compartments across the full range of flexion is summarized in Table 7.

**TABLE 6:** Means and standard deviations of the relative axial rotation of the femoral component determined from the distal and frontal points during weight-bearing lunge for the 5C® LATic, Healthy and 3DKnee cohorts.

​		Distal points					Frontal points				
		5C® LATic (°)	Healthy (°)	Effect size (p-value)	3DKnee (°)	Effect size (p-value)	5C® LATic (°)	Healthy (°)	Effect size (p-value)	3DKnee (°)	Effect size (p-value)
**Knee flexion angle (°)**	0	0	0	-	0	-	0	0	-	0	-
	10	0.4 ± 0.5	2.6 ± 2.3	1.27 (0.06)	1.0 ± 1.2	0.62 (0.31)	1.3 ± 0.9	−0.2 ± 5.2	0.40 (0.68)	−0.0 ± 3.5	0.53 (0.12)
	20	**0.5 ± 1.0**	**3.9 ± 4.2**	1.11 (0.04)	1.8 ± 1.7	0.89 (0.09)	1.4 ± 1.9	1.9 ± 3.8	0.19 (0.65)	−0.7 ± 5.1	0.54 (0.41)
	30	**0.8 ± 1.7**	**4.7 ± 5.9**	0.88 (0.04)	2.7 ± 2.0	0.96 (0.08)	2.4 ± 2.6	3.0 ± 5.1	0.15 (0.76)	−0.6 ± 6.8	0.59 (0.46)
	40	1.4 ± 2.0	5.0 ± 7.0	0.71 (0.10)	3.5 ± 2.5	0.93 (0.11)	3.4 ± 3.2	4.9 ± 5.1	0.36 (0.62)	−0.1 ± 8.9	0.52 (0.38)
	50	2.0 ± 2.4	4.2 ± 7.3	0.41 (0.32)	4.4 ± 3.0	0.84 (0.11)	3.1 ± 3.9	6.2 ± 5.3	0.66 (0.20)	0.1 ± 10.1	0.40 (0.67)
	60	2.6 ± 3.0	4.3 ± 7.4	0.31 (0.54)	5.2 ± 3.6	0.80 (0.18)	3.9 ± 4.2	8.1 ± 5.7	0.84 (0.13)	0.0 ± 11.7	0.44 (0.85)
	70	3.7 ± 3.4	7.6 ± 8.2	0.62 (0.12)	7.0 ± 3.4	0.96 (0.19)	**4.9 ± 4.9**	**13.4 ± 0.9**	2.36 (0.03)	−0.6 ± 14.3	0.52 (0.63)

Bold values indicate significant differences. Effect size and p values for the respective comparison are also presented. Since the subjects achieved different levels of active knee flexion, the analysis was limited to 70° of flexion to ensure inclusion of all participants.

**TABLE 7:** Means and standard deviations of the relative axial rotation of the femoral component determined from the distal and frontal points during non-weight-bearing flexion-extension for the 5C® LATic, Healthy and 3DKnee cohorts.

​		Distal points					Frontal points				
		5C® LATic (°)	Healthy (°)	Effect size (p-value)	3DKnee (°)	Effect size (p-value)	5C® LATic (°)	Healthy (°)	Effect size (p-value)	3DKnee (°)	Effect size (p-value)
**Knee flexion angle (°)**	0	0	0	-	0	-	0	0	-	0	-
	10	0.4 ± 0.4	2.5 ± 3.8	0.78 (0.36)	0.5 ± 0.6	0.32 (0.96)	**0.8 ± 0.7**	1.1 ± 1.3	0.27 (0.92)	**0.6 ± 2.0**	1.00 (0.04)
	20	1.2 ± 0.8	4.1 ± 5.2	0.80 (0.15)	1.2 ± 1.2	0.04 (0.97)	**1.9 ± 1.0**	**3.9 ± 1.8**	1.36 (0.005)	**0.7 ± 3.3**	1.04 (0.01)
	30	2.2 ± 1.4	3.3 ± 4.9	0.29 (0.62)	2.0 ± 1.8	0.11 (0.67)	**3.8 ± 1.8**	**5.7 ± 2.2**	0.98 (0.04)	**−0.6 ± 4.6**	1.27 (0.002)
	40	3.3 ± 1.9	2.5 ± 5.5	0.18 (0.88)	2.9 ± 2.3	0.15 (0.67)	**5.6 ± 2.1**	6.7 ± 2.4	0.45 (0.48)	**−0.5 ± 6.0**	1.35 (0.001)
	50	4.3 ± 2.3	2.7 ± 5.9	0.36 (0.62)	4.0 ± 2.6	0.13 (0.79)	**5.6 ± 2.3**	6.9 ± 3.3	0.46 (0.48)	**−0.2 ± 7.4**	1.06 (0.01)
	60	5.9 ± 1.7	2.7 ± 6.6	0.66 (0.96)	5.2 ± 2.8	0.33 (0.68)	**6.3 ± 1.6**	7.3 ± 3.3	0.38 (0.70)	**−0.2 ± 9.6**	0.95 (0.04)
	70	6.9 ± 1.9	3.5 ± 6.0	0.76 (0.19)	6.5 ± 3.0	0.18 (0.68)	7.1 ± 1.6	7.8 ± 3.1	0.25 (0.94)	0.7 ± 11.0	0.82 (0.19)
	80	7.8 ± 2.2	4.7 ± 5.5	0.74 (0.22)	7.8 ± 3.4	0.01 (0.87)	8.1 ± 1.7	7.7 ± 3.3	0.14 (0.29)	2.6 ± 13.3	0.58 (0.25)
	90	8.7 ± 2.7	5.9 ± 6.9	0.54 (0.28)	9.4 ± 3.7	0.19 (0.56)	8.9 ± 2.1	8.4 ± 4.0	0.16 (0.62)	3.4 ± 14.7	0.53 (0.32)
	100	9.7 ± 3.5	8.2 ± 7.1	0.26 (0.33)	11.3 ± 4.0	0.44 (0.56)	10.6 ± 4.9	9.7 ± 3.5	0.23 (0.77)	5.1 ± 18.2	0.41 (0.79)

Bold values indicate significant differences. Effect size and p values for the respective comparison are also presented.

**FIGURE 8:**
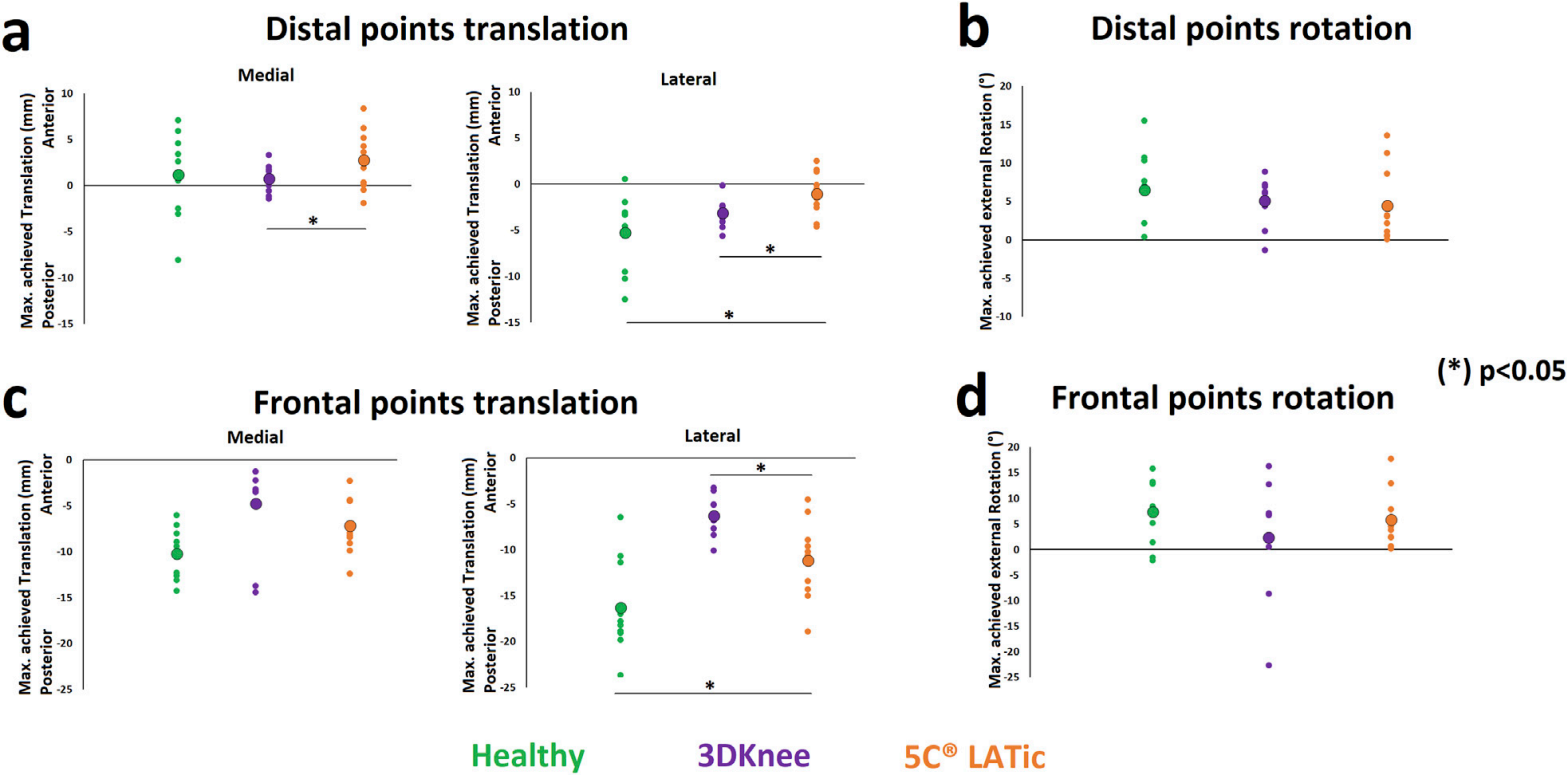
**(a)** Maximal achieved translation at maximal flexion of the distal points for each subject (large circle represents the mean value) on the medial and lateral compartments. **(b)** Maximal achieved rotation at maximal flexion derived from the distal points on the tibia plateau for each subject (large circle represents the mean value). **(c)** Maximal achieved translation at maximal flexion of the frontal points for each subject (large circle represents the mean value) on the medial and lateral compartments. **(d)** Maximal achieved rotation at maximal flexion derived from the frontal points on the tibia plateau for each subject (large circle represents the mean value). Analysis derived from the weight-bearing lunge activity.

**FIGURE 9:**
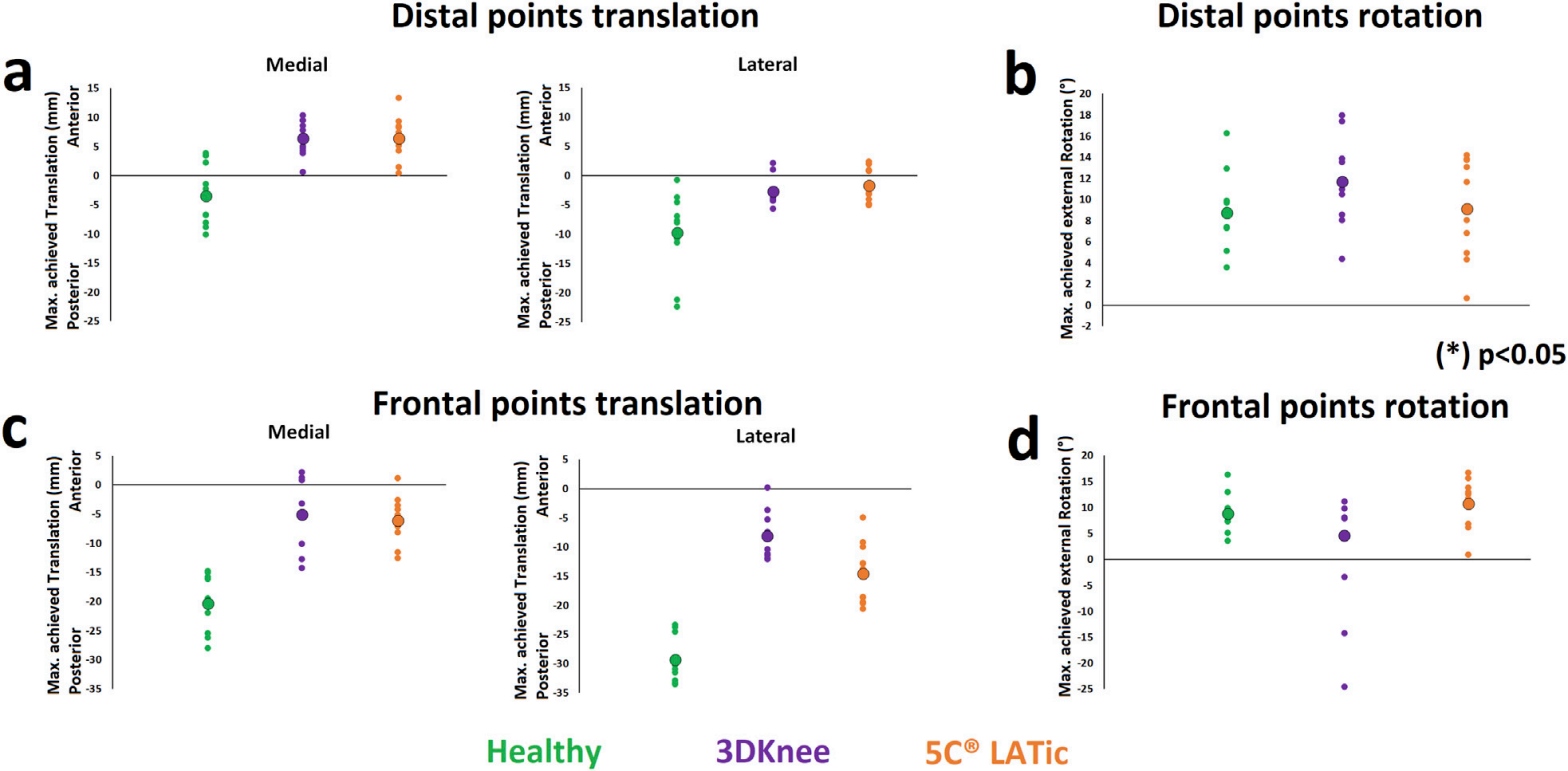
**(a)** Maximal achieved translation at maximal flexion of the distal points for each subject (large circle represents the mean value) on the medial and lateral compartments. **(b)** Maximal achieved rotation at maximal flexion derived from the distal points on the tibia plateau for each subject (large circle represents the mean value). **(c)** Maximal achieved translation at maximal flexion of the frontal points for each subject (large circle represents the mean value) on the medial and lateral compartments. **(d)** Maximal achieved rotation at maximal flexion derived from the frontal points on the tibia plateau for each subject (large circle represents the mean value). Analysis derived from the non-weight-bearing flexion-extension activity.

A graphical representation of all described movements at maximum flexion for individual patients across the three cohorts is provided in Figure 8 for the weight-bearing lunge and in Figure 9 for the non-weight-bearing flexion–extension activity.

### 3.2 Clinical data and questionnaires

In the clinical examination, all 5C® LATic patients showed improvements in all administered questionnaires compared to their preoperative state, with high overall satisfaction reported for the prosthesis. However, some inconsistencies were noted in the Forgotten Joint Score (FJS) and High-Flexion Knee Score (Table 8).

**TABLE 8:** Pre- and postoperative clinical data of the 5C® LATic cohort.

Patient	Extension (°) (pre)	Extension (°) (post)	Passive flexion (°) (pre)	Passive flexion (°) (post)	KS (pre)	KS (post)	FS (pre)	FS (post)	KSS (pre)	KSS (post)	FJS (pre)	FJS (post)	HFKS (pre)	HFKS (post)	Postop. Patient satisfaction (1–10)
1	0	0	95	120	60	94	60	90	120	184	32	82	26	42	9
2	10	0	100	120	55	100	65	100	120	200	34	84	28	43	10
3	5	0	105	125	65	90	50	90	115	180	38	79	28	41	9
4	10	0	100	115	50	85	65	90	115	175	30	64	33	36	8
5	5	0	110	135	70	100	50	100	120	200	30	80	28	44	10
6	10	5	95	115	65	80	60	80	120	160	24	32	30	33	7
7	5	0	110	130	70	100	60	100	130	200	32	84	33	41	10
8	0	0	95	135	50	100	65	100	115	200	34	82	35	42	10
9	3	0	100	125	60	90	55	90	115	180	30	75	28	40	9
10	5	0	105	115	55	85	60	85	115	170	34	60	33	35	8
11	15	5	90	95	50	70	50	65	100	135	24	28	24	28	6
Mean	-	-	100.4	120.9	59.0	90.3	58.2	90	116.8	180.3	31.1	68.1	29.6	38.6	8.7

**FIGURE 10:**
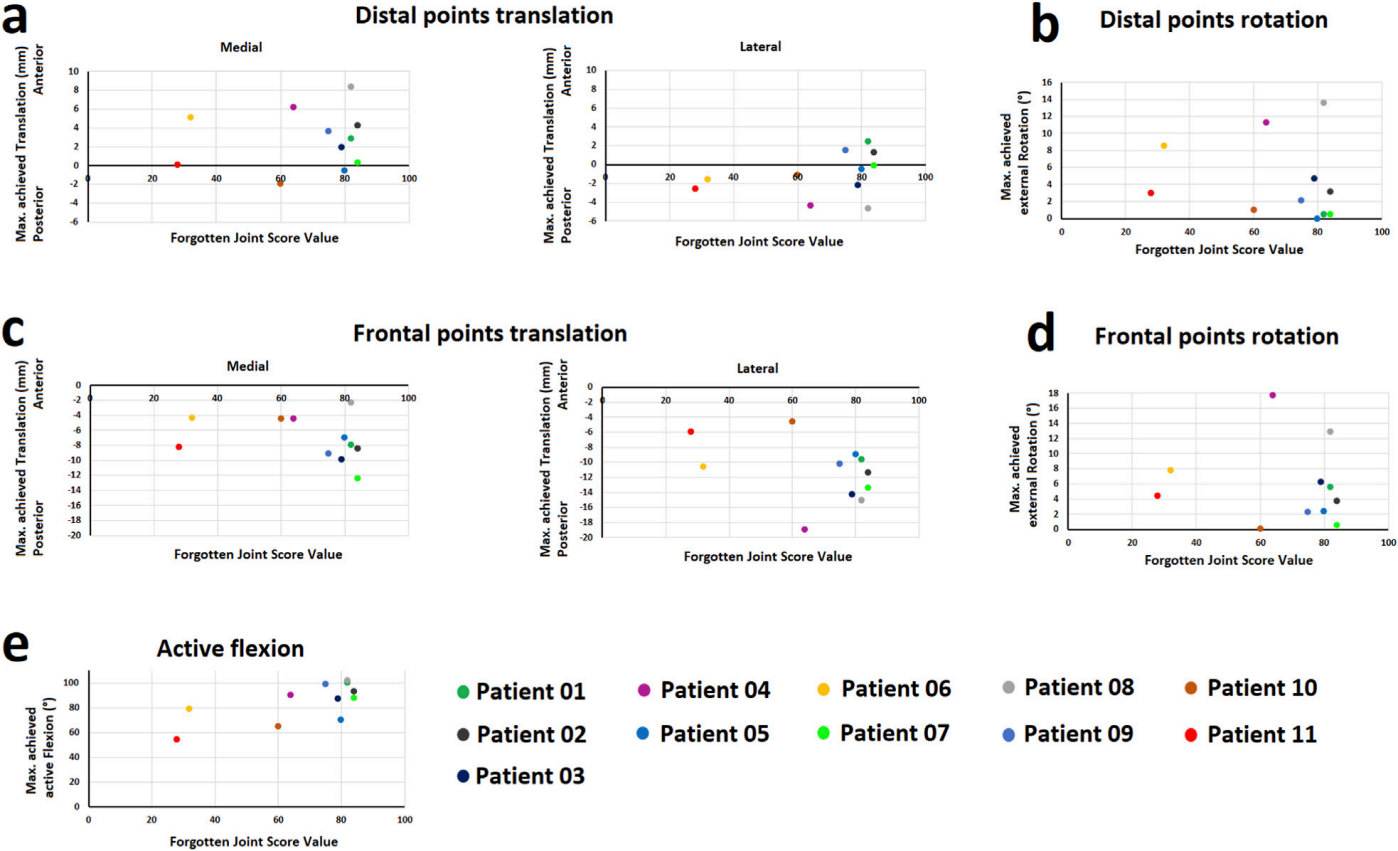
Comparison of anterior-posterior translation **(a)** and rotation **(b)** from the distal points, anterior-posterior translation **(c)** and rotation **(d)** from the frontal points and achieved flexion **(e)** during weight-bearing lunge with the Forgotten Joint Score values of each 5C® LATic patients.

A graphical representation of the kinematics during weight-bearing lunge of all 5C® LATic patients with their FJS values revealed that, in general, patients reporting high FJS values were associated with anterior translation in the medial compartment and a stable lateral compartment with additional rollback (Figure 10a). This pattern also led to clear external rotation in some cases (Figure 10b).

A similar representation using the translation of the frontal points showed that high FJS values were associated with increased posterior translation of the frontal aspect on both the medial and lateral sides (Figure 10c). A pattern that generally led to external rotation of the frontal aspect (Figure 10d).

Furthermore, in general, high FJS values were associated with greater active flexion (Figure 10e).

**FIGURE 11:**
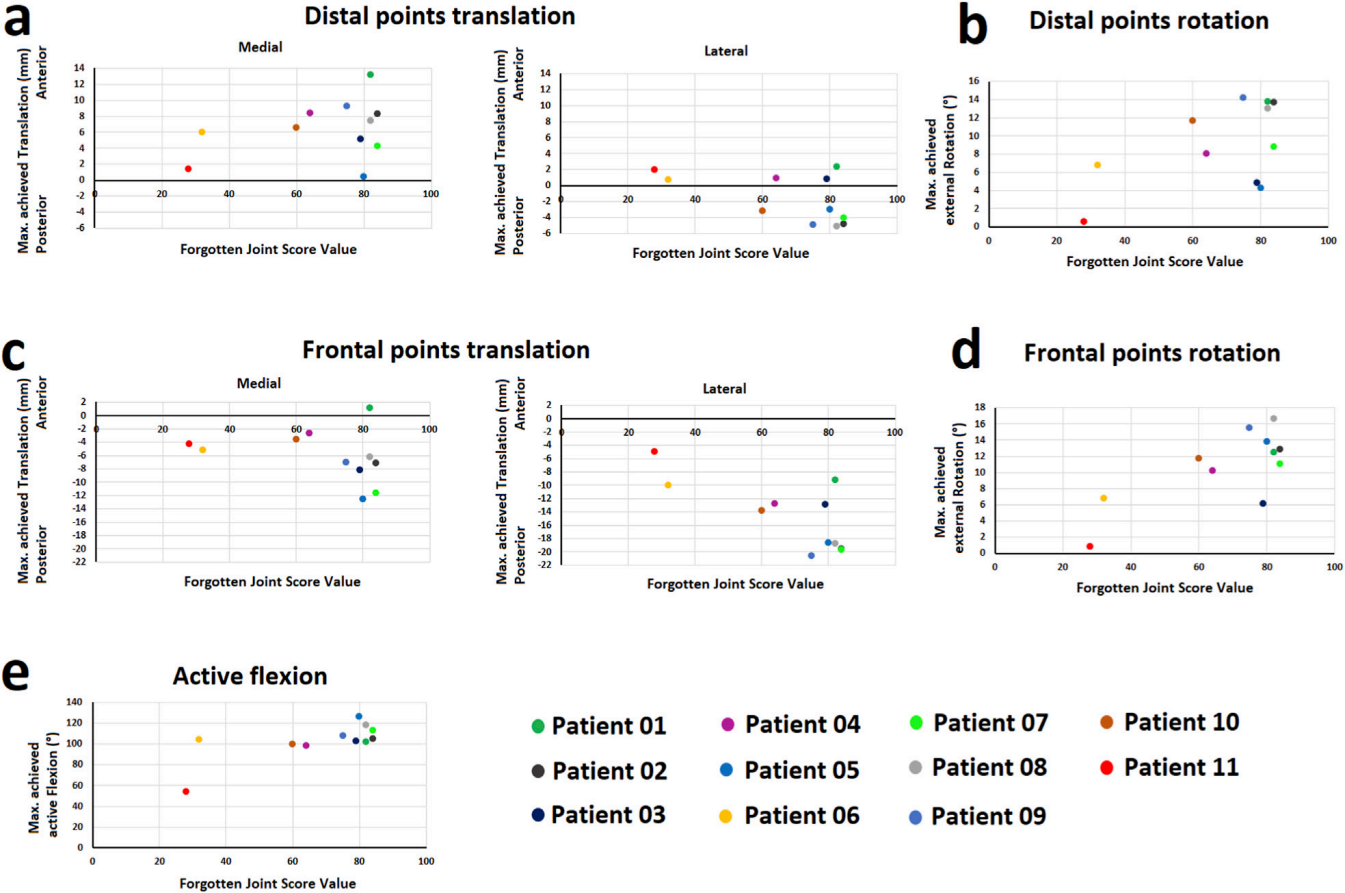
Comparison of anterior-posterior translation **(a)** and rotation **(b)** from the distal points, anterior-posterior translation **(c)** and rotation **(d)** from the frontal points and achieved flexion **(e)** during non-weight-bearing flexion-extension with the Forgotten Joint Score values of each 5C® LATic patients.

These patient-specific kinematic behaviors remained consistent even under non-weight-bearing conditions (Figure 11).

## 4 Discussion

The *in vivo* fluoroscopic analysis of the 5C® LATic TKA system demonstrated knee joint kinematics that closely aligned with the expected design-based behavior. Despite some inter-patient variability, a consistent compartment-specific pattern was observed across all analyzed subjects. The flat medial compartment enabled free movement of the medial femoral condyle, resulting in an anterior shift in all patients. In contrast, the Ball-in-socket congruency of the lateral compartment stabilized the lateral femoral condyle, as evidenced by reduced translation of the lateral distal points. Furthermore, the anterior elevation of the lateral bearing acted as a mechanical barrier and prevented excessive anterior translation, which is reflected by the absence of anterior movement of the lateral distal points (Figures 6, 7 and in Tables 2 and 3). This kinematic behavior differs from previous findings in other cruciate-retaining (CR) TKA systems, which often exhibited anterior translation in the lateral compartment (Moewis et al., 2021).

This combined compartment-specific stability in the 5C® LATic TKA system resulted in consistent axial external rotation of the femoral component during both weight-bearing lunge and non-weight-bearing flexion-extension. As shown in Figures 8, 9 and in Tables 6 and 7, the magnitude of external rotation was comparable to previously analyzed TKA systems and even to healthy cohorts. It can be assumed that this axial rotation is enabled by the combination of lateral compartment stability and medial compartment freedom, potentially supported by the sagittal design of the femoral condyles and the transition from single-to multi-radius geometry at 58° of flexion, together with the applied posterior slope between 5° and 7°.

Consistent with previous reports on knee joint kinematics under weight-bearing and non-weight-bearing conditions (Kono et al., 2019; Moewis et al., 2021; Moewis et al. 2019; Moewis et al. 2023; Pfitzner et al., 2017), this characteristic kinematic pattern became even more pronounced during non-weight-bearing flexion-extension. Importantly, the observed behavior remained largely unaffected by loading conditions, emphasizing the intrinsic stability of the implant design. Although a medial-pivot-like mechanism, as described by Konno and colleagues (Konno et al., 2014) was neither achieved nor intended, the overall kinematic behavior of the 5C® LATic system ensured that anterior translation of the lateral femoral, and thus a paradoxical motion pattern, was avoided during flexion. This mechanism may contribute to improved femoral external rotation and could lead to a more favorable distribution of contact stresses at the patellofemoral joint.

In addition, modifications of the femoral component’s frontal aspect, including 2 mm lateralization of the trochlea, widening of the sulcus angle, and flattening of the lateral frontal surface, had a direct positive influence on kinematics. This was evidenced by continuous posterior movement of the frontal femoral component throughout flexion (Figure 6; Table 4). Compared with a similar lateral-stabilized system such as the 3DKnee, the magnitude of posterior translation was significantly greater, although it still remained significantly lower than that observed in healthy knees. Nevertheless, this resulted in a clear posterior femoral position at deep flexion. This effect was also particularly pronounced in the lateral compartment during non-weight-bearing activity (Figure 7; Table 5). Although this posterior translation serves as an indirect representation of the patella movement throughout the complete flexion cycle, the selection of the frontal reference points could be valuable considering their location in the patellofemoral interaction region of the femoral component. However, direct patellofemoral kinematics could not be assessed in the present study, and therefore no causal conclusions regarding patellar tracking can be drawn. Such effects are clinically relevant considering the association of abnormal patellar mechanics with anterior knee pain (Agarwala, Mohrir, and Patel, 2013; Antinolfi et al., 2020; Duan et al., 2018; Konno et al., 2014; Lee et al., 2013; Petersen et al., 2014; van Houten, Heesterbeek, and Wymenga, 2016).

Although no direct correlation between clinical outcomes (Forgotten Joint Score, FJS) and kinematic parameters was detected (Figure 10), the data suggests a trend whereby patients with the highest FJS values exhibited the most expected tibiofemoral and frontal kinematic behavior as well as greater active flexion. For example, patient 08 (FJS: 82) demonstrated the greatest axial rotation on the tibial plateau, driven by anterior medial translation and lateral rollback, together with high posterior frontal translation and deep flexion. Similarly, patients 02 and 07 (FJS: 84) displayed stable lateral compartments, pronounced posterior frontal translation, and high flexion angles. Patient 04, despite a lower FJS of 64, also exhibited anterior medial translation, lateral rollback, and the highest posterior translation on the lateral frontal side.

In contrast, patient 11, who reported the lowest FJS score (28), showed minimal medial compartment movement and one of the lowest posterior translations of the frontal femoral component, combined with limited flexion (Figure 7). Comparable but slightly more pronounced patterns were observed in patients 06 (FJS: 32) and 10 (FJS: 60). Notably, these patient-specific kinematic behaviors remained consistent even under non-weight-bearing conditions (Figure 11), further supporting the stability of the 5C® LATic design concept.

Here we present, for the first time to our knowledge, a conceptual development approach that identifies an unmet medical need based on real *in vivo* kinematic measurements from small patient cohorts implanted with specific designs. Comparative analysis against healthy knee kinematics allowed definition of an intended use, which translated into specific design consequences. The impact of these features could then be validated through *in vivo* kinematic evaluation of patients performing identical activities using the same analytical methodology.

This study could therefore be considered a proof-of-concept investigation of a design-driven implant development strategy. As already mentioned, the primary objective was to determine if the intended design modifications resulted in the expected *in vivo* kinematic performance and not to demonstrate clinical superiority over available and certified TKA systems.

This study has limitations. The sample size was small, all patients were recruited from a single center, surgeries were performed by one surgeon and no direct patellofemoral kinematic measurements could be conducted. Furthermore, the minimum follow-up of 12 months may be early for final conclusions regarding long-term kinematic behavior. Only two activities were analyzed due to fluoroscopic and X-ray radiation related limitations, and preoperative fluoroscopic kinematic data were unavailable, preventing direct pre–post comparisons.

Nevertheless, the consistency between observed *in vivo* kinematics, clinical trends, and design-based expectations highlights the value of incorporating insights from fluoroscopic knee joint analyses into the development of future TKA systems, particularly within emerging Medical Device Regulation (MDR) pathways. Moreover, analysis of the frontal femoral component movement may incorporate valuable insights to understand patellar tracking and potentially influence decisions regarding patella resurfacing, probably in the context of anterior knee pain relief.

Finally, given the multifactorial nature of TKA outcomes, implant geometry must be considered alongside surgical technique, soft tissue balancing, alignment, and component orientation. The present findings suggest that the incorporation of *in vivo* kinematic data into implant design process may facilitate the development of TKA systems with more predictable functional behavior. Future studies should therefore consider the combination of direct patellofemoral tracking measurements, larger patient cohorts and comparative clinical TKA designs to confirm these observations.

## Data Availability

The original contributions presented in the study are included in the article/supplementary material, further inquiries can be directed to the corresponding author.
